# PROTOCOL: The relationship between homework time and academic performance among K‐12 students: A systematic review

**DOI:** 10.1002/cl2.1199

**Published:** 2021-10-26

**Authors:** Liping Guo, Jieyun Li, Zheng Xu, Xiaoling Hu, Chunyan Liu, Xin Xing, Xiuxia Li, Howard White, Kehu Yang

**Affiliations:** ^1^ Evidence‐Based Medicine Center, School of Basic Medical Sciences, and Evidence‐based Social Sciences Research Center, School of Public Health Lanzhou University Lanzhou China; ^2^ School of Higher Education Lanzhou University Lanzhou China; ^3^ Evidence‐based Social Sciences Research Center, School of Public Health Lanzhou University Lanzhou China; ^4^ Campbell Collaboration New Delhi India

## Abstract

This review will synthesize the results from publications focused on homework time and academic performance, and estimate the relationship between the two. Our objectives are: (1) To identify the extent of the relationship between homework time and students' academic performance; (2) To analyze the differences in the effectiveness of homework time across genders, grades, subject and regions; and (3) To identify the potential factors that affect homework time, such as academic subject, task difficulty, type of homework, mode of homework, parental involvement, and feedback on homework.

## 1 BACKGROUND

### 1.1 Description of the condition

Homework is defined as “any task assigned by schoolteachers intended for students to carry out during non‐school hours” (Cooper, 1989). This definition explicitly excludes (a) in‐school guided study; (b) home study courses delivered through the mail, television, audio or videocassette, or the internet; and (c) extracurricular activities such as sports and participation in clubs (Cooper et al., 2006). With the development of technology, web‐based homework has become more popular among teachers, with online platforms such as Blackboard, WebCT (www.webct.com), Homework Service (https://hw.utexas.edu/bur/overview.html), WebWorK, Study Island, and PowerSchool (Mendicino et al., 2009), as these allow students to do their homework online, and teachers to give feedback to students immediately (Callahan, 2016; Lucas, 2012; Mendicino et al., 2009). Therefore, in this systematic review, homework also includes online tasks performed outside the school.

The purpose of homework can be divided into instructional and noninstructional objectives (Lee & Pruitt, 1979). The most common instructional purpose of homework includes review, preview, and extension (Becker & Epstein, 1982; Lee & Pruitt, 1979; Mulhenbruck et al., 1999). The review assignments mainly offer the students an opportunity to practise newly acquired skills or review material learnt in class. The preview assignments introduce new skills or materials to help students prepare for unfamiliar knowledge before the class (Mulhenbruck et al., 1999), and the extension assignments involve the transfer of previously learned skills to new situations (Cooper et al., 2006; Lee & Pruitt, 1979). The noninstructional purpose of homework varies. It can be used to form better study habits, increase the students' sense of responsibility, enhance awareness of independent learning, and build communication between parents, children, and teachers (Becker & Epstein, 1982; González et al., 2001; Lee & Pruitt, 1979; Mulhenbruck et al., 1999; Van Voorhis, 2003). Homework can also be used to punish students (Epstein & Van Voorhis, 2001).

Homework is a common and widespread educational activity for many students across the world. As an achievement of the educational excellence movement, the level of homework was generalized, which was supported by the parents at the beginning. However, as homework increased more and more, parents and scholars realized the burden of homework on students. They complained that the students lost their childhood and called for less homework (Gill & Schlossman, 2003). Similarly, in the United Kingdom homework became common in the mid‐19th century, and was a matter of much debate in the 1880s as levels of homework increased in response to the introduction of payment by results for teachers and other factors (Hallam, 2004).

Recently, the World Health Organization (WHO) found that students feel the most pressured by the amount of homework (WHO, 2016). Meanwhile, parents continued to complain about excessive homework assigned to their children (Gill & Schlossman, 2003, 2004; Jerrima et al., 2019; Xue & Zhang, 2019).

Homework is often argued to improve academic performance. However, the relationship between homework and academic performance has been debated for more than one hundred years (Cheema & Sheridan, 2015; Cooper, 1989; Cooper et al., 2006; Kitsantas et al., 2011; Kralovec & Buell, 2000; Trautwein, 2007). Although several meta‐analyses of the relationship between homework and performance have found a positive correlation between homework time and academic performance (Baş et al., 2017; Cooper, 1989; Cooper et al., 2006; Fan et al., 2017), it is difficult to establish causality. More academically inclined students, who get better grades regardless, may complete their homework more conscientiously. Conversely, students who are doing badly may study harder at home to catch up.

Still, it may also be the case that the effect is not linear. Some evidence has shown that academic performance increases with the increase in homework time, but begins to decline when homework time exceeds the optimal amount of time (Ackerman et al., 2011; Krzysztof et al., 2018; Reteig et al., 2019). Based on data from the National Center for Education Statistics (NCES) survey of 58,000 high school students in grades one and two, Keith (1982) found that for anyone with any level of ability, increasing the amount of homework will improve their performance. Homework plays a compensatory role; however, the amount of homework cannot be increased indefinitely, only moderately. If it exceeds a certain limit, it will lead to a decline in performance (Keith, 1982).

### 1.2 Description of the intervention

The intervention is the homework assigned by schoolteachers for nonschool hours, and completed independently by students without additional teaching, such as online tasks and activities in study club. The comparison condition is different time spent on the homework, and we plan to divide the comparisons into several groups, such as 0–15, 16–30, 31–45, 46–60, 61–90, 90–120 min, and more than 120 min. Any type of homework will be included, such as written, oral, or practical homework. We excluded homework allocated by other people such as parents or teachers from extracurricular schools, and in‐school guided study, home study courses, and extracurricular activities such as sports and participation in clubs were excluded. Homework related to psychotherapy was also outside our definition of homework.

### 1.3 Conceptual framework

**Figure 1:**
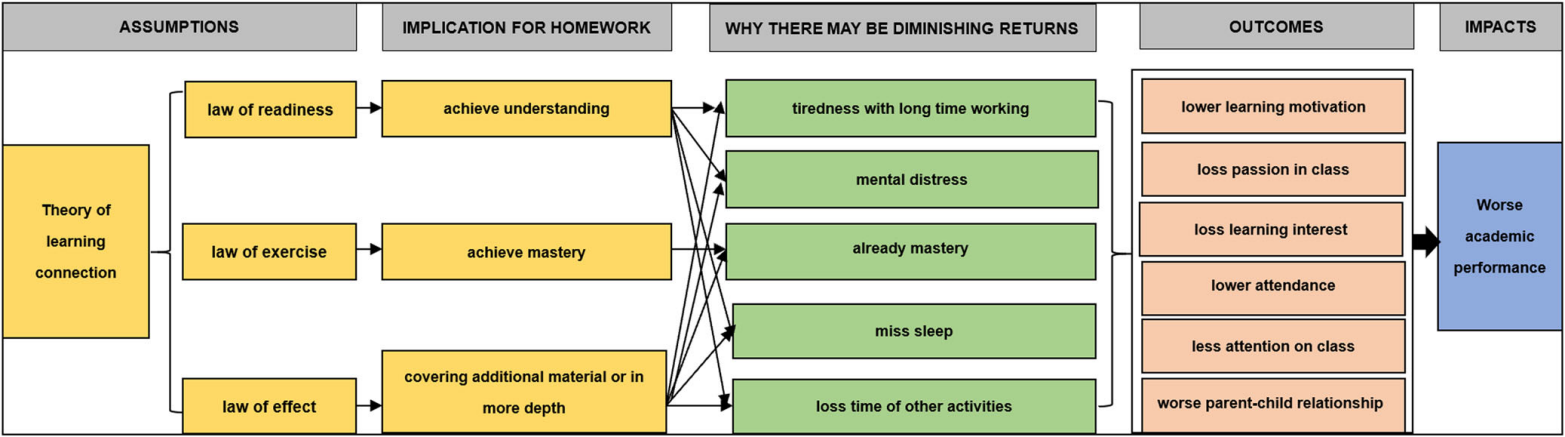
Conceptual framework for intervention and outcomes of homework

The conceptual framework for this review is the theory of change that describes how homework may affect academic performance. Figure 1 below demonstrates the conceptual framework through which the interventions are hypothesized to lead to the intended outcomes.

As in Figure 1, the law of readiness reveals that before commencing a certain learning activity, if the learners do well in the preparatory stages (including physiological and psychological) related to the corresponding learning activities, they can grasp the learning content more rapidly (Muhammad, 2015). Second, the law of exercise suggests that for a certain kind of connection formed by learners, the correct repetition of this action in practice will effectively enhance this connection. And, third, the law of effect indicates that all kinds of positive or negative feedback that learners get in the process of learning will strengthen or weaken the connection that learners have formed in their mind.

However, there are limits to our ability to stay focused, and when students spend too much time on homework, their cognitive load and mental fatigue increase, and they will feel tired and even lead to mental distress, such as anxiety, which may reduce rather than improve readiness.

Furthermore, if the purpose of homework is to achieve mastery or covering additional material, then when the content of homework has been fully understood by a student, doing more homework won't help. That is there are diminishing returns to the time spent on homework, which can reach zero.

Finally, if the students miss sleep because of long homework hours, they may be tired in school and so do worse in class or tests. Hence readiness is reduced, and the law of effect undermined as the student is not in a good condition to receive feedback or perform well.

In addition, a student's development should be multifaceted, while if they spent too much time on homework, they will lose time to take part in other activities which can contribute to their overall development.

### 1.4 Why it is important to do this review

Several systematic reviews have explored the effectiveness of homework in improving students' performance, but all the conclusions were based on the assumption of a linear correlation between homework time and performance, and none of them considered the impact of homework time on students' autonomous motivation. A summary of the evidence by Hallam (2004) makes no recommendation on the time spent on the homework. The UK Education Endowment Foundation's toolkit entry for homework for secondary school students notes quality is more important than quantity but gives no explicit recommendation on the amount of time that should be spent on homework.

Existing reviews leave the important practical question of homework time unanswered.

In 1989, Cooper conducted a review related to the relationship between homework and performance. The results showed that the average correlation for students in primary school, middle school, and high school between the amount of homework and performance was nearly *r* = 0; for students in middle school, it was *r* = .07; and for high school students, it was *r* = .25 (Cooper, 1989). In 2006, Cooper et al. (2006) conducted another systematic review to explore the effectiveness of homework to improve academic performance. The results showed that the correlation between homework time and performance for high school students was still 0.25, but for middle school students, it was nearly 0. However, they did not look explicitly at homework time.

All the above studies assumed that the correlation between homework and performance was linear, that is, that either more or less homework was better. Indeed, their reported effect size is the correlation coefficient, which is a measure of the linearity of a relationship. While homework can be a dull task that requires full mental effort, and there are limits to our ability to stay focused. Therefore, it is important for teachers, school managers, parents, and children themselves to establish the optimum duration of homework to improve its effectiveness.

### 1.5 The contribution of this review

Regardless of its aims of preparation, practice, extension or application, homework can be an effective means to improve student's academic achievement. Previous reviews indeed testify to the effectiveness of homework in relation to academic performance. More is not always better, and is restricted by students' ability to maintain their attention for a long time. The present systematic review plans to divide the participants into several groups according to the amount of time spent on homework, such as 0–15, 16–30, 31–45, 46–60, 61–90, 90–120 min, and more than 120 min to compare the test scores of different groups to identify the extent of the relationship between homework time and students' academic performance. We aim to investigate the role of homework in academic achievement, and to determine the optimum homework time by comparing the differences in outcomes between different groupings of homework time. This will be helpful for teachers and parents to better understand the role and utility of homework, and provide theoretical support for teachers to arrange homework.

## 2 OBJECTIVES

This review will synthesize the results from publications focused on homework time and academic performance, and estimate the relationship between the two. Our objectives are:

1. To identify the extent of the relationship between homework time and students' academic performance;
2. To analyze the differences in the effectiveness of homework time across genders, grades, subject and regions; and
3. To identify the potential factors that affect homework time, such as academic subject, task difficulty, type of homework, mode of homework, parental involvement, and feedback on homework.

## 3 METHODS

### 3.1 Criteria for considering studies for this review

#### 3.1.1 Types of studies

We will include treatment‐control group design or a comparison group design studies, to adequately address the effect of differing homework time on the academic performance of K‐12 students.

Randomized controlled trials (RCTs) that aim to explore the effect of homework time on academic performance by comparing the test score differences between different groups before and after the intervention will be included. In addition, non‐RCTs such as cohort studies (NRCTs), controlled before and after studies, and interrupted time series studies also will be included if they explicitly take homework as intervention, and report the time spent on homework and the mean and standard deviation of academic achievement.

Relevant correlational studies without mean and standard deviation of academic achievement will be excluded. Other study designs such as case studies, narrative reviews, and nonprimary studies such as editorials, book reviews, commentaries, and letters to the editor, will also be excluded. Qualitative evidence is also beyond the inclusion criteria in the present systematic review.

#### 3.1.2 Types of participants

This review will include studies of K‐12 school students. We excluded children with disabilities, since the context and effects may be different for that population group. Students from special education schools are excluded. If the primary study includes mixed samples (e.g., special education and nonspecial education students), we will use the sub sample excluding special needs students if reported.

#### 3.1.3 Types of interventions

In this review, we will explore the relationship between homework time and academic performance by comparing the academic scores with different amounts of time spent on homework. The eligible intervention studies must be clear that the intervention is homework assigned to students to complete during nonschool hours regularly by schoolteachers which aims to improve academic achievement. This does not mean that the intervention must consist of academic activities, but rather that the explicit expectation must be that the homework, regardless of the nature of the homework content, will result in improved academic performance. Furthermore, we will only include school‐based interventions, that is, homework allocated by other people such as parents or teachers from extracurricular schools, study clubs, and extracurricular activities such as sports and participation in clubs are excluded. Homework related to psychotherapy will also be excluded.

The comparison condition is different time spend on the homework, and we plan to divide the comparisons into several groups, such as 0–15, 16–30, 31–45, 46–60, 61–90, 90–120 min, and more than 120 min.

#### 3.1.4 Types of outcome measures

The objective of the review is to explore the impact of homework on students' academic outcomes. We will extract the homework time and academic performance provided in the primary study. The homework time is the exact time or a time frame reported by students or parents. Academic performance will be measured by the teacher, exam results and/or by the research team using any valid standardized test and reported as test scores.

As valid standardized tests, we will consider norm‐referenced tests (e.g., Gates‐MacGinitie Reading Tests and Star Math), state‐wide tests (e.g., Iowa Test of Basic Skills), national tests (e.g., National Assessment of Educational Progress). If it is not clear from the description of outcome measures in the studies, we will use electronic sources to determine whether a test is standardized or not.

#### 3.1.5 Primary outcomes

The primary outcome is academic performance (test score and standard deviation), and studies that have measured academic performance (and homework time) will be included.

#### 3.1.6 Secondary outcomes

Academic motivation and quality of homework will be included as secondary outcomes.

### 3.2 Search methods for identification of studies

#### 3.2.1 Electronic searches

The following databases will be searched from inception to present:

- Social Sciences Citation Index (Web of Science)
- ScienceDirect (https://www.sciencedirect.com/)
- Taylor & Francis Online Database (https://www.tandfonline.com/)
- The Campbell Library (https://www.campbellcollaboration.org/better-evidence.html)
- ERCI (EBSCOhost)
- EBSCO (http://search.ebscohost.com/)
- JSTOR (https://www.jstor.org/)
- PsychArticles (ProQuest)
- PsychInfo (EBSCOhost)
- ProQuest Dissertations (https://www.proquest.com/index)
- OCLC FirstSearch (https://firstsearch.oclc.org/)

Below, the search strategy for Web of Science is provided:

#1 TI = homework OR AB = homework

#2 TI = home‐work OR AB = home‐work

#3 #1 OR #2

#4 TS = K‐12 OR TS = preschool student* OR TS = pre‐school student* OR TS = Kindergarten

student* OR TS = middle school student* OR TS = high school student* OR TS = senior school

student* OR TS = primary school student* OR TS = pupil OR TS = schoolchild OR TS = junior

high school student* OR TS = school‐age

#5 TS = achievement OR TS = performance OR TS = grade OR TS = score OR TS = academic

achievement* OR TS = GPA OR TS = academic performance

#6 #3 AND #4 AND #5

#### 3.2.2 Searching other resources

We will consult the following sources of gray literature, and search the websites of organizations devoted to the education research, to identify relevant unpublished studies and reports. The following gray literature resources will be searched with the keyword “homework”:

- What Works Clearinghouse (https://ies.ed.gov/ncee/wwc/)
- Education Endowment Foundation (https://educationendowmentfoundation.org.uk/)
- European Educational Research Association (http://www.eera-ecer.de/)
- American Educational Research Association (http://www.aera.net/)
- Best Evidence Encyclopedia (http://www.bestevidence.org/)
- Open Grey (http://www.opengrey.eu/)

We will also search the Google Scholar with the keyword “homework,” and we will stop scan if there are 5 consecutive pages with no relevant studies.

The following international journals will be hand searched for relevant studies with the keyword “homework”:

- American Educational Research Journal
- Educational Psychologist
- Learning and Instruction
- Journal of Educational Research
- Journal of Educational Psychology
- Journal of Research on Educational Effectiveness
- Journal of Experimental Education.

Additionally, the primary studies included in the previous systematic reviews on the relationship between homework and academic performance will be scanned, and the reference lists will also be searched. Furthermore, the studies of experts in the research of homework (such as Cooper Harris, Trautwein Ulric, and Xu jianzhong) will be searched systematically to check our search strategy, and they will be contacted to help identify other relevant studies if possible.

### 3.3 Data collection and analysis

#### 3.3.1 Selection of studies

The selection of studies will be performed independently by the first two reviewers (Guo LP and Jieyun Li) in Rayyan (https://rayyan.qcri.org/). All titles and abstracts of the records identified after retrieval will be screened, the potentially relevant references will be located with full‐text, and the primary studies that meet our criteria will be included for further analysis. Studies that meet the selection criteria and have the outcomes of interest measured, but do not report these outcome data, will be included and described in the results section of the review. Any discrepancies between the two reviewers will be resolved by consensus with another reviewer involved (Kehu Yang). The whole process of study screening will be based on the PRISMA guidelines (Moher et al., 2009).

#### 3.3.2 Data extraction and management

Information extraction and coding will consist of two parts. The first is general information, including the information of primary study (publication, the year of publication, and the year of data collection), sample characteristics (e.g., sample size, gender, grade level, region, family economic status, parental education level), methodological characteristics (e.g., sampling method, measures of homework time, and the measure of academic performance), and the intervention characteristics (e.g., subject, mode of homework and the type of homework). The other is the effect size, including the homework time and the test score (The details are shown in Supporting Information Annex 1). This process will be conducted independently by two authors (Zheng Xu and Xing Xin), and disagreements between coders will be resolved by discussions with another author (Xiuxia Li).

- If a study contains multiple interventions (e.g., different homework modalities such as online vs. book‐based), the reviewers will only extract data that are eligible for this review.
- For academic performance, means, standard deviations (or information to estimate standard deviations), and the number of participants in each group will be extracted. If more than one measure is reported, we will extract all of them and analyze the measurement method as a moderator.
- For the homework time, we will extract the homework time interval reported in the primary studies and code the data as presented, either categorical or continuous. We will then create a continuous variable measure data set (using the mid‐point for data reported in categorical form) and at least two categorical data sets. The multiple categorical data sets will be used to test for sensitivity to the chosen thresholds, and then calculate the mean and standard deviations in each group. If the weekly homework time is reported instead of the daily homework time, we divide the total homework time by 5, and if the homework time is in hours, we convert it to minutes.

In case of controlled before and after studies, mean or median change from baseline scores will be extracted or computed by the reviewers if all necessary data are available. If change scores are not available or cannot be computed, post‐intervention values will be extracted by the reviewers.

#### 3.3.3 Assessment of risk of bias in included studies

For RCTs, the Cochrane bias risk tool will be used to assess the quality of the method and potential defects (Higgins & Green, 2011). For nonrandomized studies (including cohort studies, controlled before and after studies, and interrupted time series studies), the risk of bias in nonrandomized studies of interventions (ROBINS‐I) will be used to check the quality of the individual study (Sterne et al., 2016). In addition, the grade will be used to rate the overall quality of the evidence included in this review, ranging from high, moderate, low, and very low, based on the assessment to study design, imprecision, inconsistency, indirectness, and publication bias (Atkins et al., 2004; Schünemann et al., 2013). The risk of bias assessment will be conducted by the two authors (Zheng Xu and Xing Xin), and any disaccord will be solved by discussion with another author (Xiuxia Li).

#### 3.3.4 Dealing with missing data

If there are any missing data, we will contact the author at least twice to obtain more information if the correspondence address is available. If these data are unavailable, we will only analyze the available data, and the studies with missing data will be described in the Results section. Besides, the potential impact of missing data on comprehensive estimates will be considered in the Discussion section.

#### 3.3.5 Assessment of heterogeneity

Forest plots will be inspected to visually investigate overlaps in the confidence intervals (CIs) of the results of the individual studies. The *χ* ^2^ test will be performed, and the *Q* statistics, *I* ^2^ and *τ* ^2^ index will be adopted to evaluate heterogeneity across studies. For *Q* statistics, a *p* value of .05 will be used as a threshold for statistical significance. The *I* ^2^ index refers to the truly observed variation ratio (Borenstein et al., 2009), and 25%, 50%, and 75% of the *I* ^2^ indicate low, medium, and high heterogeneity (Higgins & Thompson, 2002). And the parameter *τ* ^2^ is the between‐studies variance (the variance of the effect size parameters across the population of studies), that is, the variance of true effect sizes (across an infinite number of studies).

#### 3.3.6 Assessment of reporting biases

Reporting bias, also called publication bias, refers to the potential of the studies with statistically significant findings to be accepted for publication, whilst those with statistically nonsignificant findings would hardly be accepted for publication. These studies would have a higher probability of being left in the “file drawer,” according to the so‐called “file drawer problem.” If 10 or more studies were identified, visual funnel plots and Egger's test of funnel plot symmetry were performed to evaluate potential publication bias (Rosenthal, 1979). If there is evidence of funnel plot asymmetry from a test, we will attempt to conduct the comparisons between the effect with that after trim and fill. The possible reasons for this (e.g., nonreporting biases, poor methodological quality leading to spuriously inflated effects in smaller studies, true heterogeneity, artefactual, and chance) will also be considered (Page et al., 2019).

#### 3.3.7 Data synthesis

We will include all intervention studies that meet our inclusion criteria and extract the mean (M) and SD of test scores. We will take the standard mean difference (SMD) and CI as our effect size index. For subgroups, we plan to divide the outcomes into several groups by time spent on the homework, such as 0–15, 16–30, 31–45, 46–60, 61–90, 90–120 min, and more than 120 min, and then compare the difference of SMD between groups to explore the role of homework time on academic achievement.

If two or more studies are identified that have investigated the effect of homework time on academic performance with sufficiently available data, a random‐effects meta‐analysis will be performed to estimate the comprehensive effect due to the expected heterogeneity between groups using the Review Manager 5 software. The pooled estimates will be presented in forest plots. If quantitative synthesis is not suitable, narrative synthesis will be adopted.

#### 3.3.8 Planned moderators

If statistically significant heterogeneity is detected, subgroup analyses with stratification analysis will be conducted to explore the source of heterogeneity based on the available data.

- Gender. Previous studies showed that girls more frequently reported managing their homework than boys (Mau & Lynn, 2000; Xu, 2007), and zero‐time homework students are most often male (Hagborg, 1991). Therefore, it is worth identifying the influence of gender on homework time and academic performance.
- Grade level. Existing reviews on homework suggest that the relationship between homework and performance is mediated by grade level (Baş et al., 2017; Cooper, 1989; Cooper et al., 2006; Fan et al., 2017). Thus, we include the grade level as a potential factor that moderates the linkage of homework time and academic performance.
- Region. Numerous studies indicated that there are regional differences in homework policies and practices (Chen & Stevenson, 1989; Tam & Chan, 2009; Zhu, 2015). The samples involved in the primary study on homework are from different regions (e.g., the United States and Asia); thus, we included the sampling region as a potential moderator in the present review.
- Publication year: Education systems are susceptible to influence of societal changes; thus, publication year is likely to be a moderator, as the homework time may change systematically over time (Cooper et al., 2006; Gill & Schlossman, 2004; Twenge et al., 2004). Thus, publication year may also potentially affect the effect sizes of homework time on performance.
- Mode of homework. With web‐learning popularized in education, online homework is being adopted by more teachers, and several researchers have argued about the effects of online homework compared to traditional homework (Callahan, 2016; Elias et al., 2017; Jonsdottir et al., 2017; Mendicino et al., 2009). In the present review, we will explore whether the relationship between homework time and academic performance is affected by the mode of homework.
- Type of homework. Teachers typically assign different kinds of homework according to their purpose. Such as reading story to parents, writing math exercises, and trying scientific experiments. In this review, we will divide the academic achievement into three groups: oral homework, paper homework and practical homework and explore whether the relationship between homework time and academic performance is different depending on the type of homework.
- The measure of academic performance: The methods used in previous homework studies included standardized tests and unstandardized assessments (Fan et al., 2017). Several studies suggested that the influence of homework on performance is larger with unstandardized assessments than with standardized tests (e.g., Cooper et al.,^1998^); therefore, there is a need to consider the measure of performance as a potential moderator.
- Subject. Several studies showed that time and effort input in homework varies depending on the subject (e.g., Trautwein, 2007; Trautwein & Lüdtke, 2009), and it is reasonable to suspect that homework may play a different role in different subjects (e.g., Cooper et al., 2006; Fan et al., 2017; Paschal et al., 1984).

#### 3.3.9 Sensitivity analysis

In addition to the implicit sensitivity analysis in both the analysis of heterogeneity and subgroup analysis, the “One‐leave‐out” method is adopted for sensitivity analysis to check for outliers that potentially influence the overall results, and test the robustness of the meta‐analysis.

## CONTRIBUTIONS OF AUTHORS

Liping Guo drafted the protocol, and all authors reviewed the draft and approved the final version.

## DECLARATIONS OF INTEREST

All authors declare no potential interest.

## INTERNAL SOURCES

This review is supported by funding of the Major Project of the National Social Science Fund of China: Research on the Theoretical System, International Experience, and Chinese Path of Evidence‐based Social Science.

## Supporting information

Supplementary Information
